# SUBCUTANEOUS ONLAY LAPAROSCOPIC APPROACH (SCOLA) FOR VENTRAL HERNIA AND RECTUS ABDOMINIS DIASTASIS REPAIR: TECHNICAL DESCRIPTION AND INITIAL RESULTS

**DOI:** 10.1590/0102-672020180001e1399

**Published:** 2018-12-06

**Authors:** Christiano Marlo Paggi CLAUS, Flavio MALCHER, Leandro Totti CAVAZZOLA, Marcelo FURTADO, Alexander MORRELL, Mauricio AZEVEDO, Luciana Guimarães MEIRELLES, Heitor SANTOS, Rodrigo GARCIA

**Affiliations:** 1 Department of Surgical Clinic and Mini Invasive Surgery - Jacques Perissat Institute, Positivo University, Curitiba, PR, Brazil;; 2 Celebration Health Florida Hospital, Celebration, FL, USA;; 3 Service of General Surgery, Hospital de Clínicas, Federal University of Rio Grande do Sul, Porto Alegre, RS, Brazil;; 4 Service of General and Laparoscopic Surgery, Pitangueiras Hospital, Jundiaí, SP, Brazil;; 5 Service of General Surgery, Einstein Hospital, São Paulo, SP, Brazil;; 6 Service of General Surgery and Digestive System, Mandaqui Hospital, São Paulo, SP, Brazil;; 7 Service of General Surgery and Trauma, Santa Maria Health House, Barra Mansa, RJ, Brazil;; 8 Digestive Surgery Service, Americas Medical Services / Pro-Cardiac Hospital, Rio de Janeiro, RJ, Brazil;; 9 Department of Digestive System Surgery, Municipal Public Server Hospital, São Paulo, SP, Brazil.

**Keywords:** Hernia, Laparoscopy, Diastasis, muscle, Hérnia, Laparoscopia, Diástase muscular

## Abstract

**Background:**

Diastasis of the rectus abdominis muscles (DMRA) is frequent and may be associated with abdominal wall hernias. For patients with redudant skin, dermolipectomy and plication of the diastasis is the most commonly used procedure. However, there is a significant group of patients who do not require skin resection or do not want large incisions.

**Aim:**

To describe a “new” technique (subcutaneous onlay laparoscopic approach - SCOLA) for the correction of ventral hernias combined with the DMRA plication and to report the initial results of a case series.

**Method:**

SCOLA was applied in 48 patients to correct ventral hernia concomitant to plication of DMRA by pre-aponeurotic endoscopic technique.

**Results:**

The mean operative time was 93.5 min. There were no intra-operative complications and no conversion. Seroma was the most frequent complication (n=13, 27%). Only one (2%) had surgical wound infection. After a median follow-up of eight months (2-19), only one (2%) patient presented recurrence of DMRA and one (2%) subcutaneous tissue retraction/fibrosis. Forty-five (93.7%) patients reported being satisfied with outcome.

**Conclusion:**

The SCOLA technique is a safe, reproducible and effective alternative for patients with abdominal wall hernia associated with DMRA.

## RESUMO

**Racional:**

A diástase dos músculos retos abdominais (DMRA) é frequente e pode estar associada à presença de hérnias da parede abdominal. Para pacientes com excesso de pele, a dermolipectomia e plicatura da diástase é o procedimento mais comumente utilizado. Entretanto, há um grupo significativo de pacientes que não necessitam ressecção de pele ou não desejam grandes incisões.

**Objetivo:**

Descrever uma “nova” técnica (Subcutaneous Onlay Laparoscopic Approach - SCOLA) para a correção das hérnias ventrais combinada à plicatura da DMRA e relatar os resultados iniciais.

**Métodos:**

A técnica SCOLA de correção de hérnia ventral concomitante com a plicatura da DMRA por técnica endoscópica pré-aponeurótica foi aplicada em quarenta e oito pacientes.

**Resultados:**

O tempo operatório médio foi de 93,5 min. Não houve nenhuma complicação intra-operatória e nenhuma conversão. Seroma foi a complicação mais frequente (n=13, 27%). Apenas um (2%) apresentou infecção de ferida operatória. Após seguimento médio de oito meses (2-19) apenas um (2%) paciente apresentou recidiva da DMRA e um (2%) retração/fibrose do tecido subcutâneo. Quarenta e cinco (93,7%) relataram estarem satisfeitos com resultado.

**Conclusão:**

A técnica SCOLA é alternativa segura, reprodutível e efetiva para pacientes com hérnia da parede abdominal associada à DMRA.

## INTRODUCTION

Diastasis of the Rectus Abdominis Muscles (DMRA), defined as distancing from the muscular borders in the midline greater than 2.2 cm, is not a rare condition^3^. It is characterized by bulging in the anterior wall of the abdomen when the patient exerts contraction of the abdominal musculature and/or increase of the intra-abdominal pressure, being often confused with hernia of the abdominal wall. DMRA is usually not associated with symptoms, pain or discomfort, as well as any risk of complications^12^ ^,^ ^25^. The main complaint is aesthetic, a buldging at the abdomen, making its treatment, nowadays, performed by plastic surgeons.

However, the simultaneous presence of a hernia of the anterior abdominal wall is not uncommon^6^ ^,^ ^23^. In this scenario, general surgeons are usually called upon to perform the repair, and they do not always take into account the particularities associated with greater abdominal wall weakness due to DMRA. The concomitant presence of it and the non-use of meshes appear to be the most important factors associated with failure to repair midline defects and consequent recurrence of the hernia^21^ ^,^ ^23^.

Treatment of DMRA, associated or not with abdominal wall hernias, in patients with excess skin is usually performed by a large transverse incision in the lower abdomen associated with dermolipectomy^1^ ^,^ ^17^ ^,^ ^28^. Plication techniques are the most commonly used and may or may not be associated with mesh placement. However, there is a group of patients in whom there is no need for skin resection, in which conventional operation with midline longitudinal incisions results in unfavorable results from the aesthetic point of view^15^ ^,^ ^20^.

In order to improve these results, in the 1990s, the first alternatives of DMRA correction with the use of endoscopic techniques without the need for large cutaneous incisions were described^9^ ^,^ ^30^. The conventional laparoscopic technique of intraperitoneal mesh placement does not seem to solve the problem of diasthesis unless it is also repaired by intracorporeal or transfascial sutures. Nevertheless, the results have been questionable and extraperitoneal alternatives have been described^4^ ^,^ ^24^.

The aim of the present study was to describe a “new” technique (SCOLA) for the correction of ventral hernias combined with the plication of the diastasis of the rectus abdominis muscles and present the initial results of a series of cases. 

## METHODS

Between October 2015 and October 2017, 48 patients were submitted to the correction of ventral hernia concomitant with the plication of DMRA by pre-aponeurotic endoscopic technique. The repair was indicated for patients presenting with primary abdominal or incisional hernias with concomitant diastasis of the rectus abdominis muscles. Exclusion criteria were: contraindication for general anesthesia, previous history of abdominoplasty, coagulopathy, cirrhosis of the liver or renal insufficiency. Patients with abnormally non-midline hernias, those with no desire for concomitant diastasis correction or those with indication/desire for correction of excess skin were also excluded.

### Technique

Antibiotic prophylaxis was routinely used, consisting of administration of 1 g intravenous cefazolin in anesthetic induction. The patient is placed in dorsal decubitus, under general anesthesia, with a slight extension of the hip and the legs abducted. The surgeon is placed between the patient’s legs and the assistant laterally.

**FIGURE 1:**
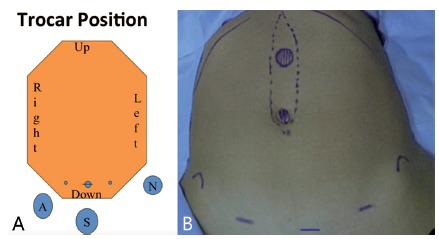
A) Schematic illustration of the positioning of the surgical team and positioning of the portals (S - surgeon; A - assistant); B) photo with a representative picture of the main hernial defects (umbilical and epigastric) and the DMRA and positioning of the portals

**FIGURE 2:**
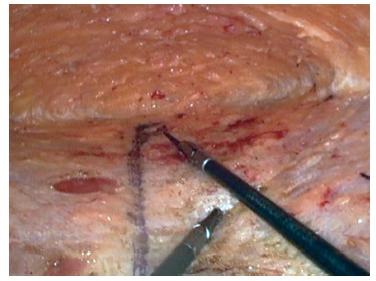
Superior dissection until subcostal region and lateral extension

**FIGURE 3:**
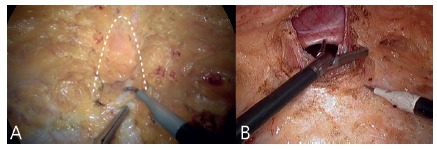
A) Hernia sac projection (dotted line); B) dissection/ressection of the hernia sac and its content

**FIGURE 4:**
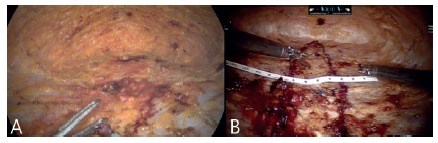
A) Full dissection; B) diastasis marking (blue) e ruler measurements

**FIGURE 5:**
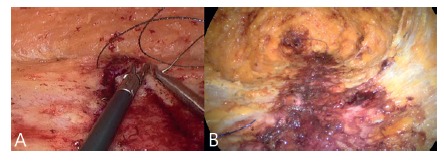
A e B) DMRA plication

**FIGURE 6:**
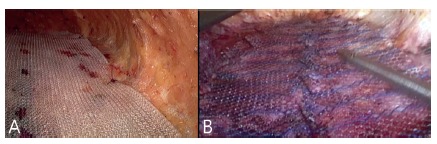
A e B) Mesh convering DMRA plication in a pre-aponerotic position (onlay)

A small 2 cm transverse incision just above the pubis is performed (equivalent to the C-section incision). The subcutaneous tissue is dissected until it reaches the anterior aponeurosis of the rectus abdominis muscle. With the aid of Farabeuf type retractors the subcutaneous tissue is separated from the anterior aponeurosis with monopolar cautery both superior and laterally to create sufficient space in the placement of a portal of 11 mm for optic by the previous incision and two auxiliary 5 mm laterally (Figure 1A and B). A purse-string suture is performed in the suprapubic incision both to fix the portal of the optic and to prevent CO2 leakage. The CO2 insufflation pressure is maintained at 8-10 mmHg. With the use of a grasper and a hook or scissors connected to the electrocautery, the subcutaneous tissue is dissected from the anterior aponeurosis of the rectus abdominis muscle. The umbilicus is disinserted from the aponeurotic muscle plane and the dissection progresses until it reaches the xiphoid medially and the ribs laterally. In the latero-lateral direction the dissection should be at least 12-15 cm (Figure 2). Hernial sacs are found as projections from the aponeurotic muscle plane toward the upper subcutaneous tissue (usually containing the preperitoneal fat). The hernia sac is easily dissected and the contents reduced to the abdominal cavity (Figure 3A and B). If the peritoneum is opened and consequent pneumoperitoneum develops, it does not appear to interfere with the operative field. At the end of the dissection, it is easy to identify the hernia defects, as well as the diastasis of the rectus abdominis muscles (Figure 4A and B). The next step is proper correction of the diastasis and the hernia defect through a suture continues to approach the edges of the right and left abdominal rectus muscle in the midline. The suture should extend from the xiphoid to at least 2-3 cm below the umbilicus. The use of barbed sutures may facilitate this step and appears to allow better closure at the midline (Figure 5A and B). The use of mesh to reinforce the repair was at the discretion of the surgeon, depending on the size of the hernia/DMRA defect. After plication, in the cases where it was chosen, a polypropylene mesh is introduced in the craniocaudal direction from the xiphoid to the 3-4 cm below the umbilicus with lateral overlap of at least 3-4 cm (Figure 6A and B) . The mesh can be fixed with tackers, suture or glue. The umbilicus is fixated back to the musculoaponeurotic plane through one or two simple sutures. Due to the subcutaneous dissection, a closed drain is introduced in this space using the same cutaneous incision of the portal of 5 mm. To decrease the seroma, fixation of the subcutaneous tissue can be performed to aponeurotic plane, although difficult technically. The alternative for this suture is the use of glue in this space, allowing adhesion of the subcutaneous tissue to aponeurotic/mesh plane and consequent reduction of dead space.

## RESULTS

### Patients’ characteristics

SCOLA for the correction of ventral hernias concomitant with DMRA plication was performed in 48 patients, 28 women and 20 men, with a mean age of 44.25 years (32-61). The mean BMI was 27.7 kg/m^2^ (between 22-32). Thirty-one (64.5%) had a single defect, while the remaining 17 (35.5%) had two or more hernias. Primary hernias represented 79% of cases (n=38) while 10 (21%) had recurrent hernias. The mean hernia size was 2.3 cm (1.5-4 cm) while the mean DMRA size (longest distance) was 4.05 cm (3-6).

### Operative results

Four cases were operated with the aid of a robotic platform and the other 44 (91.6%) by conventional laparoscopy. The mean operative time was 93.5 min (70-150). In only three cases, a mesh was not used in association with plication and defect closure. There were no conversions to open procedure. There were no intraoperative complications and bleeding was negligible in all cases.

### Postoperative complications

Fifteen patients (31.2%) had complications. Seroma was the most frequent (n=13, 27%). Seroma was reabsorbed without intervention in seven, while in six puncture was necessary for drainage. Of these, three patients needed only one puncture while one needed multiple and the other two drainage open - one associated with dermolipectomy. After a mean follow-up of eight months (2-19), one (2%) patient presented recurrence of DMRA and one (2%) retraction/fibrosis of the subcutaneous tissue. Forty-five (93.7%) patients reported being satisfied with the outcome of the surgical treatment. Only one (2%) had surgical wound infection and was treated with antibiotic therapy.

## DISCUSSION

DMRA is a frequent condition, especially during pregnancy and regresses spontaneously after delivery in most women. However, up to a third still present DMRA 12 months postpartum^27^. DMRA is characterized by thinning and widening of the alba line generally combined with sagging abdominal wall muscles. DMRA is defined according to Beer’s classification: distance between the straight abdominal muscles greater than 2.2 cm, measured 3 cm above the belly button with relaxed abdomen^3^.

Most patients with DMRA are treated conservatively, as it is usually not associated with symptoms or risk of complications^12^ ^,^ ^25^. Physiotherapy programs have been used for patients with DMRA; however, the results presented are not encouraging^18^ ^,^ ^22^. The strengthening of the abdominal muscles seems to play some role only adjuvant to the surgical treatment^29^.

The main complaint in patients with DMRA is alteration of the shape of the abdomen. Surgical repair is generally considered an aesthetic procedure, and is addressed to plastic surgeons.

However, thinning of the linea alba is an important risk factor for the development of abdominal wall hernia^15^. The concomitance of hernias in patients with DMRA is not uncommon. In these cases, patients usually seek the general surgeons for repair of the abdominal hernia, and the diagnosis of associated DMRA is performed.

Although there is no standard method for the repair of anterior abdominal wall hernias and the use of meshes is questioned, recent studies have shown results that support its routine use, even in small hernias^2^ ^,^ ^10^. Arroyo et al^2^, in a randomized study, demonstrated a significant reduction in the risk of recurrence (1% vs. 11%, p=0.0015) without an increase in the rate of complications when polypropylene mesh was used. A prospective cohort - Danish Ventral Hernia Database^10^ (n=4786) - also showed a reduction in the risk of relapse from 5.6% to 2.2% (p=0.001) when mesh was used for repair. The rate of complications was similar to repairs based on suture alone. In this study, although the size of the defects was small (mean=2.3 cm, between 1.5 and 4 cm) in 93.7% of the cases mesh was used.

If the use of meshes is more accepted, it seems that surgeons have neglected the importance of taking into account the concomitant presence of DMRA for the treatment of midline hernias. It is true that concomitant treatment of diastasis involves more complex operations, and patients do not always desire or need such correction. However, even though DMRA is not a cause for complaint, the weakening it causes in the midline - and in cases around the hernia defect - has been associated with a high risk of relapse. Kohler et al^21^ reported a 31.1% relapse in patients with epigastric or umbilical hernias smaller than 2 cm with DMRA vs. 8.3% without DMRA after 24 months follow-up. The non-use of mehs, absorbable sutures and the concomitant presence of diastasis have been reported as the main technical factors related to relapse^2^ ^,^ ^10^ ^,^ ^21^.

Several options for the concomitant treatment of abdominal hernia associated with DMRA have been described, from open, laparoscopic, hybrid or endoscopic techniques^8^ ^,^ ^9^ ^,^ ^30^. Regardless of the form of access, it seems that the plication techniques have greater acceptance in the literature since the opening of the midline is associated with the potential greater risk of incisional hernia and its complications.

For patients with excess skin, additional dermolipectomy offers better results. However, a significant number of patients do not have excess skin or do not want a large incision in the lower abdomen. Similarly, a longitudinal incision in the upper abdomen for plication of diastasis and correction is associated with disappointing esthetic results. In these cases, alternative is the endoscopic correction of DMRA through a 4-5 cm incision using the C-section incision if previously existing or corresponding and another peri-umbilical incision^13^. Hybrid technique that associates the endoscopic vision with the use of instruments of conventional operation, in which the pre-aponeurotic space is dissected to the xiphoid followed by plication of the diastasis.

Recently this procedure was described by “exclusive” endoscopic technique, that is, through small suprapubic incisions for the portals and CO2 insufflation to maintain the operative field^4^ ^,^ ^24^. However, this article presents some technical modifications in relation to the original study published by Argentine surgeons, mainly the placement of a larger screen in a pre-aponeurotic position and absence of relaxation incisions, a technique we call SCOLA. The main advantages are to reduce the complications of surgical wound and the aesthetic result. In this initial series, only one patient (2%) had a superficial wound infection treated conservatively and 93.7% reported being satisfied with the results obtained. The indication was for patients without obesity or excess skin (mean BMI=27.7, between 22 and 32)

Another alternative is endoscopic-assisted reconstruction of the linea alba, known among others as ELAR^20^. It is a hybrid technique that, from a peri-umbilical incision with extension 2-3 cm higher and endoscopic vision aid, the pre-aponeurotic space is dissected until the xiphoid and after, plasturing the diastasis reinforced by the placement of polypropylene mesh (ELAR plus). Several authors have reported satisfactory results with this technique, despite complications related to operative wound in up to 6.4%^19^ ^,^ ^20^.

The main complication of these techniques is postoperative seroma described in up to one third of patients. In this series, 27% presented seroma clinically diagnosed. However, as reported by most authors, it tends to reabsorb spontaneously (53.8% in this series) and is considered a minor complication. Here again, half of the seromas resolved after the first puncture while, in the other three patients, one required three punctures, one open drainage and another undergoing dermolipectomy in evolution.

Although not accurately assessed in this study, there seems to be a correlation between the length of drainage and the occurrence of seroma. Patients with drainage for up to two weeks, or drainage less than 20 ml day, had lower incidence than those where it remained less than 10 days or drainage less than 50 ml day.

Alternatives to the pre-aponeurotic techniques have been described and have as main advantage to minimize the incidence of the seroma. Schwarz et al^26^ described a hybrid technique that, through a peri-umbilical incision with the aid of endoscopic vision, has the retromuscular space dissected for placement of the mesh, known as MILOS. Daes et al^11^ and Belyansky et al^5^ have described and have used totally extraperitoneal techniques for the correction of anterior wall hernias associated with DMRA. Despite very encouraging results, these procedures are more complex and require greater anatomical knowledge and laparoscopic skills than onlay techniques.

Laparoscopic plications with transfascial sutures and intraperitoneal meshes are also options of minimally invasive techniques and small occurrence of seroma. However, transfascial sutures are painful beyond the higher cost associated with intraperitoneal meshes. The closure of the defect with placement of large preperitoneal meshes to reinforce peri-hernia weakness - known as umbilical TAPP - in patients who do not need or wish to correct diastasis has recently been used^7^ ^,^ ^16^.

The results of abdominal wall hernia repair with concomitant DMRA correction reported in the literature are quite varied, according to the different techniques. In addition, there are few comparative studies, just as follow-up is generally short. In this series, despite the small follow-up time (mean=8 months, between 2 and 19), only one case (2%) of relapse was reported. Only three (6.25%) presented unsatisfactory results - a recurrence, an encapsulated seroma that resulted in dermolipectomy and a retraction/fibrosis of the subcutaneous tissue.

Some cases (n=4) of this series were operated using a robotic platform. According to the surgeons who performed under conventional laparoscopy and robotics, there were no additional advantages to those already known as better ergonomics and stability of the operative field. The work portals placed on the plication axis and mesh fixation, which are performed on the “floor” of the operative field, allow the sutures to be performed easily by laparoscopy. The impression that the authors of this paper had, despite not comparing difference in operative time, is that barbed sutures are important to aid in the plication of DMRA.

New series and long-term results are needed; however, it seems good option for patients without indication of dermolipectomy

## CONCLUSION

SCOLA technique is a safe, reproducible and efective alternative for patients with ventral hernias associated with DMRA.
